# Closure of L3 pedicle subtraction osteotomy via an open-bottom hinged table in 3D video

**DOI:** 10.3171/2020.1.FocusVid.19718

**Published:** 2020-01-01

**Authors:** Chih-Chang Chang, Praveen V. Mummaneni, Joshua Rivera, Rory Mayer, Dean Chou

**Affiliations:** 1 Department of Neurosurgery, University of California, San Francisco, California;; 2 Department of Neurosurgery, Neurological Institute, Taipei Veterans General Hospital, Taipei;; 3 School of Medicine, National Yang-Ming University, Taipei, Taiwan; and; 4 University of California, San Francisco, California

**Keywords:** pedicle subtraction osteotomy, S2AI, flat back syndrome, navigation, video

## Abstract

Iatrogenic flat back deformity generally can be treated with a pedicle subtraction osteotomy (PSO) (Chan et al., 2018; Lu and Chou, 2007). One of the difficulties with PSO is that a controlled closure can sometimes be problematic in that there may be translation of the spine, manual pushing of the spine, and significant stress on the pedicle screws, which may risk loosening. The authors present a video of their surgical technique for PSO closed by passive closure using an open-bottom hinged table. This allows the osteotomy to be closed without any force on the screws and without significant manual forces on the spinal column.

The video can be found here: https://youtu.be/pUECEjKdmSk.

## Transcript

**0:20** This is the video about closure of an L3 pedicle subtraction osteotomy via an open-bottom hinged table with 3D video. This is the patient demographic information and preoperative lateral radiographs showing the flat back syndrome. And these are the CT and MRI demonstrating the pathology.

**0:44** This is the open-bottom hinged table which will be used for closure of the osteotomy. The navigational reference arc should be placed away from the actual screw entry point because the arc itself can interfere with placement of the S2AI screws. This should be considered when planning placement of the reference arc.

**1:06** After the navigation has been registered, here is the view of sacroiliac region. Planning of the sacroiliac screws is then performed in order to align the heads with previous construct. We first start with a very small opening in order to reduce blood loss to place these screws through a mini-open approach. This is done with a drill and subsequent tap through the SI joint under navigation, and then a 90-mm screw is placed under navigation. The same is done on the other side. And this is performed through a mini-open approach while aligning the screw head with previous instrumentation for easier rod placement. The screws are stimulated in the normal fashion. A postoperative image shows good position of the screws.

**2:17** After this, the 3D video shows that the spine is now opened. The old implants have been removed and new implants are placed with the screws above and below the osteotomy buried ventral to allow for the four-rod construct. The lateral wall of the osteotomy level, in this case L3, is dissected using blunt instrumentation to dissect the segmental vessels away as well as the lumbar plexus and psoas. During this maneuver, the segmental vessels can be torn and significant bleeding can ensue. This can be stopped with GELFOAM-type products and packing, and once the bleeding has subsided, the use of bipolar in a judicious manner can also be applied for hemostasis.

**3:11** A sponge is then placed lateral to the vertebral body to clearly identify the lateral wall and to protect the segmental vessels and the lumbar plexus. Care must be taken to remove this sponge prior to closure of the osteotomy because closure can trap the sponge in the osteotomy itself. A temporary rod is also placed to prevent translation during the pedicle subtraction osteotomy. Osteotomes are then used to cut a wedge in the vertebral body, and this is done under navigation to ensure that a proper wedge is cut. The bone is removed and saved for autograft.

**4:00** First, we start with removal of the pedicle, cephalad, and caudad. And the bone is subsequently saved. After this has been done, the lateral wall can also be removed in a V-shape to ensure proper closure of the osteotomy. Navigation can be used to assess the depth of osteotomy. After that has been performed on one side, the other side is then performed with temporary rod on the side that already has had the osteotomy. This prevents translation and closure of osteotomy during the procedure.

**4:56** The osteotomy is performed in a V-shape to ensure that a wedge will be created and not a shortening of the vertebral column. The osteotomy is generally performed up to about 90% of the vertebral body. The ventral dura is then dissected and a central impactor is used to remove the final cortex.

**5:21** The osteotomy is then closed using the table as the table hinges into an extension. The osteotomy is closed with passive motion without any force on the screws or the construct. After this, the short rod is subsequently placed and further compression can be applied if necessary. The short rods are ventral to the long rods that will be placed from the upper instrumented vertebrae down to the pelvis. This constitutes the four-rod construct.

**6:06** These are the postoperative images demonstrating correction of the spinopelvic parameters. The patient’s hospital course was uneventful without complications. His hospital stay was 7 days after the osteotomy.

## Acknowledgments

Dr. Chang has received a grant from Yen Tjing Ling Medical Foundation, a nongovernment, nonprofit organization.
